# Evaluation method and design of greenhouse pear pollination drones based on grounded theory and integrated theory

**DOI:** 10.1371/journal.pone.0311297

**Published:** 2024-10-29

**Authors:** Tao Wang, Yanxiao Zhao, Leah Ling Li Pang, Qi Cheng

**Affiliations:** 1 Anyang Institute of Technology, School of Mechanical Engineering, Anyang, Henan, China; 2 Razak Faculty of Technology And Informatics, Universiti Teknologi Malaysia, Kuala Lumpur, Malaysia; University of the West of England, UNITED KINGDOM OF GREAT BRITAIN AND NORTHERN IRELAND

## Abstract

Greenhouse cultivation promotes an efficient and environmentally friendly agricultural production model, significantly enhancing resource sustainability and advancing sustainable agriculture. Traditional greenhouse pollination methods are inefficient and labor-intensive, limiting the economic benefits of greenhouse pear cultivation. To improve pollination efficiency and achieve fully automated mechanized operations, an innovative design method for greenhouse pear pollination drones has been developed. First, design criteria were extracted using Grounded Theory (GT), and the Analytic Hierarchy Process (AHP) was employed to determine the weight of user demand evaluation indicators. Next, the Quality Function Deployment (QFD) method translated user needs into technical requirements, resulting in the final ranking of design element weights. The drone was then designed based on these weighted rankings, yielding an optimal solution. This method quantifies the functional requirements of the product, effectively identifying key needs for the greenhouse pear pollination drones and proposing targeted solutions. Additionally, it provides a design reference for other highly functional agricultural machinery products.

## Introduction

Greenhouse cultivation effectively controls indoor temperature and humidity, overcoming regional limitations and significantly increasing crop yields [1,2]. The Chinese Academy of Agricultural Sciences developed the greenhouse four-season pear in 2013 as a new agricultural variety. This pear, a unique economic crop in northern China, is prized by consumers and farmers for its sweet, juicy fruit, high yield, and climate independence. However, the isolation of greenhouse crops from external climates results in a lack of natural pollinators, leading to low pollination success rates and high labor demands. Common pollination methods include bee pollination, manual pollination, mechanical spraying, and liquid pollination [3–5]. The pollination efficiency of bees reduces labor costs but poses a risk of disease transmission and is sensitive to temperature and humidity [3,6]. This makes it unsuitable for the Greenhouse in the cold winters in northern China. Due to the short blooming period of pear trees, manual pollination is necessary to ensure fruit quality and work efficiency. Traditional manual pollination in greenhouses involves using brushes, requiring significant labor [7,8]. Farmers face challenges such as poor air circulation, high humidity, and the height of the pear trees. These conditions, combined with the physically demanding nature of the task, reduce pollination rates and negatively impact farmers’ health [9]. Mechanical spraying reduces labor but cannot precisely apply pollen to flower stigmas, resulting in wastage and poor pollination outcomes [10]. Liquid pollination, which enhances stigma moisture and nutrients and extends the fertilization period, offers faster pollination [11,12]. However, the height of pear trees makes uniform pollination from multiple angles difficult. Farmers must carry heavy sprayers and use ladders, which does not fundamentally resolve the issues of high labor intensity and low efficiency.

With the rapid advancement of modern information technology and its widespread application in agriculture, traditional farming is transitioning to smart agriculture. Agricultural drones have gained increasing attention and use. Drones can quickly and non-destructively gather crop growth, yield estimation, nutrient monitoring, pest and disease surveillance, and tree canopy information through various sensors. This enables farmers to obtain reliable field information promptly, facilitating precise operational management [13]. Drones also perform agricultural tasks such as aerial spraying and seeding, adapting to diverse operational environments [14–16]. This reduces the damage to crops and soil caused by large ground-based equipment, thereby improving operational efficiency, reducing labor intensity, and promoting large-scale, intensive production [17]. In recent years, drone pollination technology has been widely used in agricultural production, particularly in the pollination of hybrid rice, with promising results [18]. Researchers like Abutalipov have demonstrated that drones can automate pollen transport, significantly enhancing pollination efficiency, though this method is complex and costly [19]. Potts and colleagues have noted that while some drone technologies can perform pollination, their efficiency remains low and does not meet the requirements for precise pollination [20]. Koşar et al. found that using drones for pollinating walnuts results in higher fruit set rates and achieves the required pollination with a reduced amount of pollen [21]. Hiraguri et al. proposed a "search pattern" method to identify pollination targets, which enables drones to precisely locate the flowers for pollination. However, this drone design is limited by its inability to automatically change batteries or replenish pollen during operation, rendering it unsuitable for extended pollination tasks and inefficient for use in large fields [22]. Although preliminary research into drone pollination technology has shown promise [23], many agricultural drones are modified commercial models [24]. There is a lack of research on drones specifically designed for pollinating tall fruit trees in the unique, enclosed environment of greenhouses. The operational environment in greenhouses demands different design parameters compared to existing pollination drones. This study proposes a new drone technology for pollinating greenhouse pear trees. This method saves labor and increases pollination success rates compared to traditional methods, making it ideal for medium to large greenhouse pear orchards.

In terms of research methods, many manufacturing companies have adopted customer-centric approaches, integrating multiple research methods into product design with notable success [25–27]. For instance, Ginting et al. explored the process of translating consumer needs into product design, highlighting the integration of AHP (Analytic Hierarchy Process) and QFD (Quality Function Deployment) in case studies [28]. Similarly, Yan et al. combined AHP to determine the weight of various user requirement indicators with QFD to establish strong correlations between user needs and product quality attributes, successfully designing agricultural drones [29]. However, with the increasing complexity of smart products, the combined AHP and QFD approach alone cannot fully meet user needs. This study builds on existing theoretical research by utilizing Grounded Theory to thoroughly capture user requirements and expert opinions, helping designers achieve optimal solutions. This method has proven effective in the field of product design [30]. Traditionally, scholars have used expert interviews and questionnaires to establish design criteria, often relying on direct induction and summary, which introduces subjectivity and potential bias into the final product design. To address the limitations of existing design methods, this study combines Grounded Theory with integrated theory to overcome these shortcomings. Grounded Theory, a bottom-up qualitative research method, does not start with theoretical assumptions. Instead, it identifies core concepts from systematically collected interview data, extracted from the experiential knowledge of industry experts [31]. This approach allows for a degree of freedom and openness, unaffected by preexisting views, and can uncover overlooked factors in current theories, thus refining the designers’ experience-based summaries scientifically and objectively [32]. In conclusion, the absence of objective design principles as a reference leads to subjective ambiguity in design activities, resulting in longer development cycles and higher costs. This study addresses these gaps by providing a novel solution for the efficient pollination drone design for greenhouse pear cultivation. It also offers designers effective theoretical guidance, filling existing research voids and enhancing product development efficiency.

## Materials and methods

This study has received ethical exemption approval from the Ethics Committee of Anyang Institute of Technology. The focus of this article is on methodological research and does not involve any studies on human organs, animal tissues, or other organisms. Participants were recruited from April 1, 2023, to April 10, 2023. All participants are adults who provided informed consent and voluntarily agreed to participate. Additionally, all collected data and information are anonymized to ensure participant privacy.

### Theoretical overview

#### Grounded theory

Grounded Theory, introduced by sociologists Glaser and Strauss in 1967 [33], is a methodology for developing theories based on empirical data. This approach involves summarizing and refining data from semi-structured interviews to develop systematic theories, extracting core concepts that reflect the essence of the phenomena studied [34]. Grounded Theory has been widely applied in current product design research, with its main contributions being the systematic exploration of user needs and the optimization of the design process [35,36]. In this study, Grounded Theory is employed to identify and understand both explicit and latent user needs, providing a scientific basis for designing products that better align with user expectations. This approach, which generates theories from actual data, ensures that the design process is highly practical and targeted, while mitigating the impact of subjective assumptions.

#### Analytic hierarchy process

The Analytic Hierarchy Process (AHP), developed by operations research expert Thomas Saaty in the 1970s, is a systematic and hierarchical evaluation method [37]. Over the past four decades, AHP has become a widely used and accepted tool for addressing complex decision-making problems in various fields [38]. The principle of AHP involves breaking down complex problems into quantifiable objectives and summarizing them using a combination of qualitative and quantitative methods. In product design, AHP assists designers in analyzing user needs and clarifying design criteria [39]. The specific steps are as follows:

Step 1: Constructing a Hierarchical Model. The decision problem is structured into a hierarchy with the goal at the top, criteria in the middle, and indexes at the bottom.

Step 2: Creating a Judgment Matrix. After establishing evaluation levels and criteria, the elements in the hierarchy are compared in pairs to form a judgment matrix C.


$$C={\left(C_{ij}\right)}_{n\times n}$$ 1

In the formula: *n* represents the number of indicators, c_ij_ represents the importance value of factors i and j relative to the target, *i*,*j* = 1,2,⋯,*n*.

Step 3: Use the geometric mean method to calculate the weights, multiply the indicators of the judgment matrix row by row, and obtain a new vector M_j_, as shown in formula (2):

$$M_{j}=\prod_{i=1}^{n}a_{ij}(j=1,2,\cdots ,n)$$ 2

In the formula: *n* represents the order of the matrix, and a_ij_ represents the elements in the judgment matrix。

Step 4: Calculate the geometric mean of each row of indicators, see formula (3):

$$a_{j}=\sqrt[{n}]{M_{j}}$$ 3

Step 5: Normalize the results and calculate the relative weight, see formula (4):

$$\omega_{j}=\frac{a_{j}}{\sum_{j=1}^{n}a_{j}}$$ 4

Step 6: Consistency test. In order to ensure the rationality and compatibility of the weight values in the judgment matrix, a consistency test is required after determining the weight values of the judgment matrix and each evaluation index. Calculate the maximum characteristic root, see formula (5):

$$\lambda_{\max}=\frac{1}{n}\sum_{j=1}^{n}\frac{{\left(A\omega \right)}_{j}}{\omega_{j}}$$ 5

In the formula: n is the number of orders of the judgment matrix; (Aω)_i_ is the i-th component of the vector.

Step 7: Calculate the consistency index *CI*, see formula (6):

$$CI=\frac{\lambda_{\max}-n}{n-1}$$

In the formula: λ_max_ indicates the maximum characteristic root of the judgment matrix; *n* indicates the order of the judgment matrix.

Step 8: Calculate the consistency ratio, see formula (7):

$$CR=\frac{CI}{RI}$$ (7)

In the formula, RI represents the random consistency index. The RI values of matrices of different orders are shown in Table 2. When CR < 0.1, the consistency test passes, otherwise it fails. It is necessary to rebuild the matrix and perform the consistency test again until it passes. The RI values are shown in Table 1.

**Table 1:** RI value of matrix order 1–9.

1	2	3	4	5	6	7	8	9
0	0	0.58	0.90	1.12	1.24	1.32	1.41	1.45

### Quality function deployment

**Fig 1:**
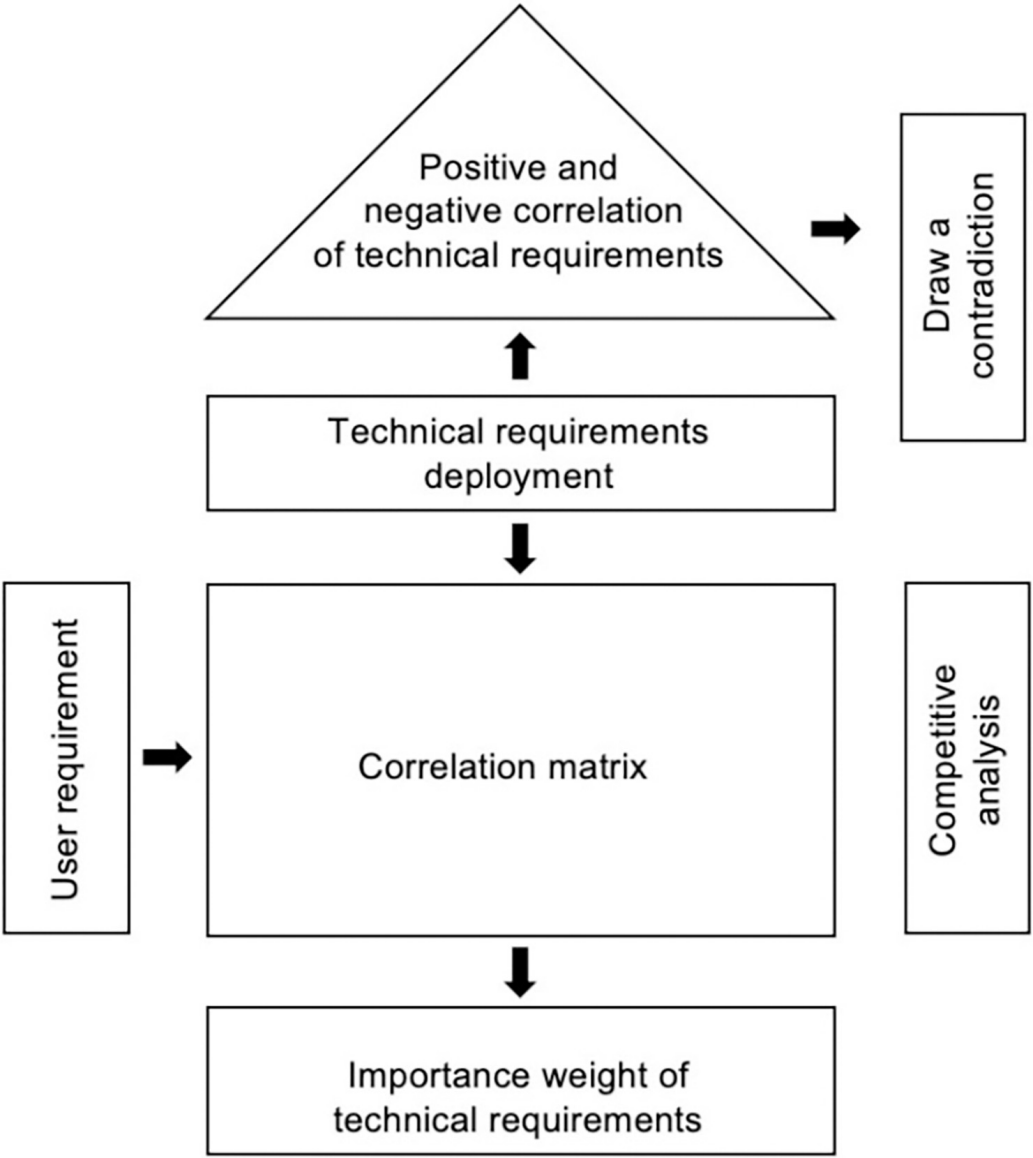
User needs hierarchy diagram.

Quality Function Deployment (QFD), introduced by Japanese quality experts Shigeru Mizuno and Yoji Akao in the late 1960s, is a customer-driven product design methodology [40]. This approach uses the construction of a "House of Quality" (HOQ) to integrate customer needs, preferences, and expectations into the product design process. By translating user requirements into technical characteristics, QFD provides a feasible method for ensuring product quality. Today, QFD is widely applied in industry, academia, and practical contexts [41,42]. It is particularly valuable in innovative design, helping designers improve research and development efficiency [43]. The key component of QFD is building the HOQ model. This model visually represents the positive and negative correlations between user expectations and technical requirements, enabling designers to effectively calculate the importance weights of various technical demands and identify conflicting requirements quickly. As shown Fig 1. In this study, the focus is on determining the weight of technical requirements for greenhouse pear pollination drones. Therefore, we will use only the relevant correlation matrix part of the QFD theory. This approach will help identify and prioritize the critical technical needs to enhance the design and functionality of greenhouse pear pollination drones.

### Design process framework

This study has been exempted from ethical review by the Ethics Committee of Anyang Institute of Technology. As a methodological research project, it does not involve any research on human organs, animal tissues, or other living organisms. Experts from relevant disciplines were invited to participate in in-depth interviews as human subjects. All participants were adults and volunteered for the study. Before the interviews, the researchers informed all participants about the purpose, process, and use of interview data, as well as their rights, and obtained written informed consent. All data were collected anonymously, ensuring that participants’ personal information remained confidential.Therefore, all research methods and procedures in this study adhere to ethical principles and regulations.

The overall process is divided into three phases: ranking user requirement indicators, translating user requirements into product functions, and resolving conflicts between functions and technical implementation.

Phase One: User Requirement Analysis. Using Grounded Theory, we conducted surveys and in-depth interviews with greenhouse pollination workers and relevant experts to identify the evaluation criteria for the pear pollination drone.

Phase Two: Hierarchical Analysis. We applied the Analytic Hierarchy Process (AHP) to establish a hierarchical model of user needs, calculating the weight of each requirement for the pollination drone.

Phase Three: Quality Function Deployment. Incorporate the user requirement weights quantified through AHP into the QFD House of Quality model. This model assessed the correlation between user requirement weights and technical characteristics, determining the importance of each technical requirement. Based on these importance rankings, we derived the optimal design solution.

**Fig 2:**
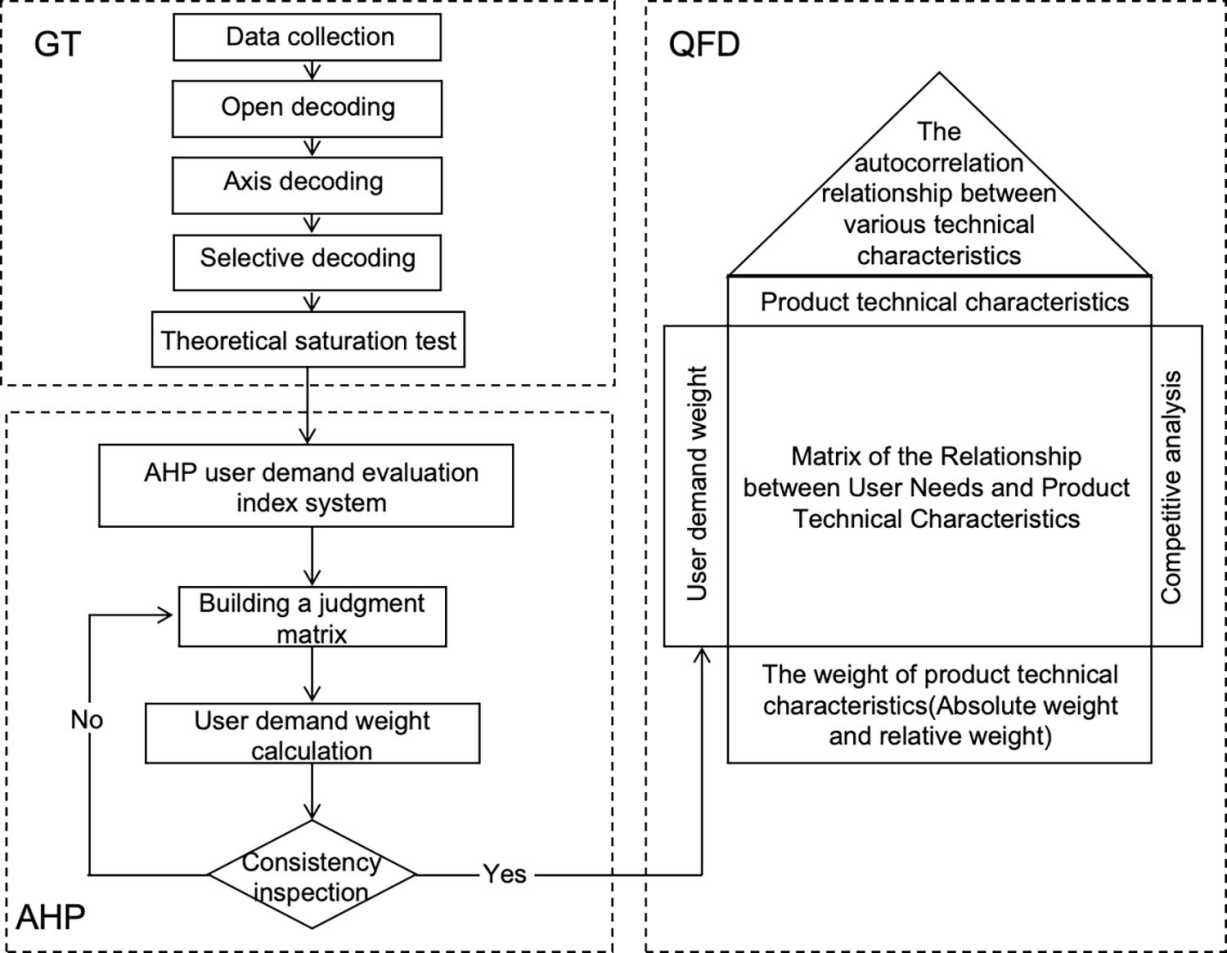
Design process framework.

This design and development process is characterized by its scientific rigor and logical coherence. It moves beyond superficial, intuitive, and subjective design practices to a method that guides product development and design with strong correlations and logical structure, as shown in Fig 2.

## Analysis and results

### Grounded theory analysis

#### Selection of expert sample

This study involves five experts with extensive industry experience. The first expert is a researcher from the Chinese Academy of Agricultural Sciences specializing in pear cultivation, who will provide insights on tree ecology and cultivation techniques. The second expert is an agricultural machinery design engineer with 13 years of experience, offering technical guidance on drone applications in agriculture. The third expert is an agricultural representative with six years of experience growing pears in greenhouses, managing over 1,000 acres in Xinjiang, China, and will provide user feedback on machinery operation. The fourth expert is an industrial designer with seven years in agricultural drone design, contributing knowledge on design, technical conversion, and latest developments. The fifth expert is a researcher with five years of experience in agricultural big data, providing insights on smart control systems, data analysis, and remote monitoring to integrate drones into smart agriculture.

#### Data collection

In-depth, semi-structured interviews were conducted with the experts. Interviewers guided the participants without leading them, ensuring each interview lasted at least 30 minutes. With participants’ consent, interviews were recorded to ensure accurate data collection. Interview questions are shown in Table 2.

**Table 2:** Semi-structured interview questions.

Expert category	Questions
Greenhouse planting technology expert	1. When formulating the design criteria for greenhouse pollination drones, what do you think is the most important technical criterion?
	2. What do you think are the main problems with the current pollination methods of greenhouse pear trees?
	3. Do you think the application of drone pollination in greenhouse pear trees is feasible? Why?
	4. When formulating the design criteria for greenhouse pollination drones, what do you think is the most important technical criterion?
	5. What are the special requirements of the environmental conditions in the greenhouse (such as temperature, humidity, and spatial layout) for the operation of drones?
	6. What are your expectations and prospects for the application of drone pollination technology in future agriculture?
Agricultural machinery design engineer	1. What do you think are the shortcomings of the existing outdoor pollination drones in greenhouse environments?
	2. What do you think are the key criteria for designing greenhouse pollination drones?
	3. What intelligent functions (such as path planning, real-time monitoring) do drones need to have to improve pollination efficiency?
	4. How to find a balance between performance and cost when designing drones?
	5. How to balance cost and performance when designing pollination drones?
	6. What is your outlook on the application of Lishu greenhouse drone pollination technology in other greenhouse crops?
Grower representative	1. What pollination methods do you use?
	2. Which pollination method do you think is more effective?
	3. What are the shortcomings of existing greenhouse pollination drones?
	4. When using a pollination drone, what functions do you most want it to have?
	5. In order to ensure the effectiveness of drones at work, what factors do you think are the most critical in the design of greenhouse pollination drones?
Industrial designer	1. What do you think are the key criteria for designing a greenhouse pear pollination drone?
	2. How do you ensure the adaptability and durability of the drone in the greenhouse environment when designing it?
	3. Based on your experience in agricultural drone design, what suggestions or innovative solutions do you have for the specific design of the greenhouse pear pollination drone? For example, the sensor technology, flight control system, etc.
	4. Can you share some of the latest developments and technological trends in the field of agricultural drone design? How do these trends affect the design and technical application of greenhouse pear pollination drones?
Agricultural big data expert	1. What types of agricultural data are particularly important for the design and operation of drone pollination?
	2. How to ensure that intelligent systems can achieve efficient data collection, analysis and application?
	3. What suggestions or innovative solutions do you have for the intelligent control system of greenhouse four-season pear pollination drones?
	4. What are your prospects for the future development of the integration of agricultural big data and drone technology?

#### Open coding

Recorded interviews were transcribed and analyzed using ATLAS for Mac. To minimize subjectivity, open coding was employed to categorize raw data, identifying new insights from phenomena observed in the data. This process involved conceptualizing raw information, summarizing repeatedly to remove redundant and irrelevant data, ultimately resulting in 11 categories, as detailed in Table 3.

**Table 3:** Open decoding process.

Original data representative sentence	Initial concept	Category
1. "Once the drone suddenly lost control, I wanted it to automatically return or land in a safe location." 2. "When the battery suddenly ran out of power, I needed an emergency shutdown mechanism to ensure that the drone would not fall on the crops." 3. "If the drone hits an obstacle, it should have an obstacle avoidance system to automatically adjust its route." 4. "Considering the crop layout and planting density in the greenhouse, the drone design needs to optimize the pollination path and method to ensure efficient pollination operations while reducing energy consumption."	1. Automatic return function 2. Emergency stop mechanism 3. Automatic obstacle avoidance function 4. Optimize flight path	Emergency response
1. "Drones can accurately locate the flowers of each tree, ensuring that pollen can be effectively spread." 2. "The optimized pollination path design can ensure that drones cover the largest area in the shortest time, improving pollination efficiency." 3. "Drones can evenly distribute pollen, avoid pollen waste, and improve the overall pollination rate."	1. Accurately locate pollination 2. Optimize pollination path 3. Efficient pollen distribution	High pollination efficiency
1. "We want to use high-quality battery materials to ensure that the battery will not explode under high temperature conditions." 2. "The drone should be equipped with a temperature monitoring system to monitor the battery temperature in real time to prevent overheating." 3. "An automatic power-off protection mechanism is required to immediately cut off the power supply when the battery temperature is too high to avoid the risk of explosion."	1. High-quality battery materials 2. Temperature monitoring system 3. Automatic power-off protection	Battery safety
1. "We hope that the drone uses high-strength materials to ensure that it remains stable during long-term use." 2. "The optimized structural design can keep the drone stable during flight and not easily damaged." 3. "The drone should have good impact resistance and be able to withstand accidental collisions without affecting its use."	1. High-strength material 2. Optimized structural design 3. Impact resistance	Stable structure
1. Greenhouses usually have limited space, so drones should be designed to be of moderate size, able to flexibly pass through the narrow space between crops, while ensuring a safe operating distance and efficient pollination coverage. 2. "We hope that drones are lightweight, easy to carry and operate." 3. "If drones can be folded, storage and transportation will be more convenient." 4. "Drones should be designed to be easy to carry and store, so that users can easily bring them to the fields."	1. Lightweight design 2. Folding structure 3. Easy to carry and store	Good portability
1. "We need the drone to be equipped with powerful LED lights for clear illumination at night." 2. "The drone should have an automatic light sensing system that can automatically adjust the lighting according to the ambient light intensity." 3. "When working at night, we hope that the lighting angle of the drone can be adjusted to suit different work needs."	1. Safety warning light 2. Automatic light sensing system 3. Adjustable lighting angle	Lighting function
1. "We need drones to be able to transmit data in real time so that we can understand their working status at any time." 2. "Drones should be equipped with high-speed communication modules to ensure that information can be quickly conveyed to operators." 3. "When problems occur, drones should be able to issue an alarm immediately so that we can take timely measures."	1. Real-time data transmission 2. High-speed communication module 3. Instant alarm system	Timely information feedback
1. "The user interface of the drone should be designed to be simple and easy to understand, so that operators can quickly get started." 2. "I hope the drone has a one-button start and stop function to reduce complicated operation steps." 3. "We need intuitive operation instructions so that users can easily complete various operations without tedious training." 4. "Adding an automatic pollination function can greatly reduce the difficulty of manual operation." 5. "Automatic measurement, adaptive pollination, and 24-hour operation."	1. Easy-to-understand user interface 2. Intuitive operation guide 3. Automatic cruise and obstacle avoidance pollination	Intelligent operation
1. "I hope that the drone can work normally in extreme high and low temperature environments." 2. "The drone should be waterproof and dustproof and can be used in rainy days and dusty environments." 3. The environment in the greenhouse is usually quite special, and the drone needs to be waterproof, dustproof, and corrosion-resistant to cope with the effects of high humidity, temperature changes, and chemical pesticide residues. 4. There may be signal interference or weak signals in the greenhouse. The drone’s communication system should have stable data transmission capabilities to ensure real-time monitoring and the accuracy of operating instructions.	1. High and low temperature resistance 2. Waterproof and dustproof 3. Strong data transmission capability	Strong environmental adaptability
1. “Drone design should choose bright orange or yellow colors to improve visibility inside the greenhouse.” 2. “The color design should have high contrast to ensure that the drone can stand out against the complex crop background.” 3. “The drone’s color scheme should be easily identifiable at night or in low-light environments for easy operation and monitoring.”	1. Bright appearance color, such as orange or yellow. 2. High-contrast design to stand out against complex backgrounds. 3. Color scheme that can be easily recognized in low-light environments.	Easy to identify
1. "The drone design should integrate simple quick-release devices and standard connectors so that operators can quickly disassemble and assemble" 2. "The use of lightweight materials and modular design can help reduce weight and simplify the assembly and disassembly process of the drone." 3. "In order to improve operational efficiency and safety, the design should take into account the convenience of manual operation and ensure that the disassembly process is simple, fast and reliable."	1. Simple disassembly and assembly interfaces and connection parts. 2. Quick release devices and standardized connectors. 3. Lightweight materials and modular design.	Easy to disassemble and assemble

#### Axial coding

Axial coding involved organizing raw data into related categories and identifying major categories based on logical relationships. The process distilled data into three main categories: functionality, human-machine interaction, and safety, as shown in Table 4.

**Table 4:** Axial coding process.

Main category	Subcategory	Connotation
Functionality	High pollination efficiency	Design drones to ensure efficient pollen dispersal rates and precise pollination operations.
	Lighting function	Integrate powerful lighting systems to ensure effective operation and monitoring in low-light conditions.
	Strong environmental adaptability	Able to adapt to a variety of greenhouse environments, including changes in temperature, humidity and crop types.
	Easy to disassemble and assemble	Simple modular connection device for quick deployment and maintenance.
Human-computer interaction	Timely information feedback	Integrated real-time data transmission and feedback system enables operators to obtain key information instantly.
	Portability	Lightweight design, easy to carry and move.
	Intelligent operation	Simple operation, set one-click hosting function to reduce user learning cost.
Security	Easy to identify	Bright color schemes are used to improve visibility and recognition in complex environments.
	Battery safety	Equipped with a highly safe and stable battery system to avoid safety risks caused by battery problems.
	Sturdy structure	Ensure that the drone structure is strong and durable and can operate stably for a long time.
	Emergency response	Integrate emergency response mechanisms and automatic obstacle avoidance technology to improve resilience and safety in emergencies.

#### Selective coding

Selective coding extracted the most critical core categories by analyzing the relationships between major categories and integrating them into a coherent framework. This method provided a comprehensive summary of category interrelations, as detailed in Table 5.

**Table 5:** Selective coding process.

Typical path relationships	Nature of relationship	Connotations
Safety → User satisfaction → Design solution	Intermediary relationship	Whether the safety is reliable will affect user satisfaction and thus affect the design plan
Functionality → User satisfaction → Design solution	Intermediary relationship	Whether the functionality is reflected will affect user satisfaction and thus affect the design plan
Human-computer interaction → User satisfaction → Design solution	Intermediary relationship	Whether human-computer interaction will affect user satisfaction and thus affect the design plan

#### Theoretical saturation testing

Theoretical saturation testing ensured the validity and reliability of the research model. By re-analyzing three reserved data sets until no new concepts or categories emerged, we confirmed the theoretical saturation of the model.

#### Hierarchical model construction

Using expert interviews and the Analytic Hierarchy Process (AHP), we organized and categorized user requirements for greenhouse pear pollination drones into three levels: goal layer, criteria layer, and solution layer. The first layer (goal layer) represents the overall user demand for greenhouse pear pollination drones (A). The second layer (criteria layer) divides user requirements into functionality (B1), human-machine interaction (B2), and safety (B3). The third layer (solution layer) further breaks down these criteria into specific needs, as shown in Table 6. This hierarchical model sets the stage for subsequent matrix analysis.

**Table 6:** Hierarchical model.

Target layer	Criteria layer	Indicator layer
Greenhouse pear pollination drones (*A*)	Functionality (*B*_1_)	High pollination efficiency
		Lighting function
		Strong environmental adaptability
		Easy to disassemble and assemble
	Human-computer interaction (*B*_2_)	Timely information feedback
		Intelligent operation
		Simple operation
	Security (*B*_3_)	Eye-catching colors
		Explosion-proof batteries
		Sturdy structure
		Emergency response

### User demand analysis based on AHP

#### Calculating user requirement weights

After constructing the user requirement hierarchy, we used the Analytic Hierarchy Process (AHP) to build a judgment matrix for user needs. This approach allows for hierarchical analysis of complex problems with multiple objectives, followed by decision consistency validation to determine user requirement weights, thus minimizing decision bias. The calculation steps are as follows:

Step 1: Construct the Judgment Matrix. We invited the five experts mentioned earlier to compare the pollination drone’s requirement criteria pairwise, creating the judgment matrix. The comparison used a 1–9 scale (see Table 7 for scale values and meanings).

**Table 7:** Judgment matrix index importance level numerical scale table.

SCALE VALUE	IMPORTANCE LEVEL	THE MEANING OF IMPORTANCE LEVEL
**1**	Equally important	Indicator X is of equal importance compared to indicator Y
**3**	Slightly important	Indicator X is marginally important compared to Indicator Y
**5**	Outstandingly important	Indicator X is significantly more important than Indicator Y
**7**	Extremely important	Indicator X is extremely important compared to indicator Y
**9**	Completely important	Indicator X is completely important compared to Indicator Y
**2, 4, 6, 8**	Eclectic use	The importance level is between two adjacent importance levels
**1/2,1/3. . .1/9**	Anti-comparison	If the importance scale value of indicator X over indicator Y is n, and vice versa is 1/n

Step 2: Calculate the user demand weight values using formulas (2)-(4), as shown in Tables 8–11.

Step 3: Perform a one-time test using formulas (5)-(7). The test results are shown in Table 12. The CR index is less than 0.1, which meets the consistency test.

**Table 8:** Judgment matrix and weights of each indicator requirement (*B*_1_—*B*_3_) under the target layer.

*A*	*B*_1_	*B*_2_	*B*_3_	*W*
*B*_1_	1	5	2	0.5949
*B*_2_	1/5	1	1/2	0.1285
*B*_3_	1/2	2	1	0.2766

**Table 9:** Judgment matrix and weights of each demand factor (*C*_1_—*C*_4_)under function *B*_1_.

*B*_1_	*C*_1_	*C*_2_	*C*_3_	*C*_4_	*W*_1_
*C*_1_	1	5	3	3	0.5317
*C*_2_	1/5	1	1/2	1/2	0.1223
*C*_3_	1/3	2	1	1	0.1856
*C*_4_	1/3	3	1	1	0.1856

**Table 10:** Judgment matrix and weights of each demand factor (*C*_5_—*C*_7_) under *B*_2_.

*B*_2_	*C*_5_	*C*_6_	*C*_7_	*W*_2_
*C*_5_	1	1/3	1/5	0.1096
*C*_6_	3	1	1/2	0.3091
*C*_7_	5	2	1	0.5812

**Table 11:** Judgment matrix and weights of each demand factor (*C*_8_—*C*_11_) under *B*_3_.

*B*_3_	*C*_8_	*C*_9_	*C*_10_	*C*_11_	*W*_3_
*C*_8_	1	1/3	1/5	1/7	0.0572
*C*_9_	3	1	1/4	1/6	0.1101
*C*_10_	5	4	1	1/2	0.3079
*C*_11_	7	6	2	1	0.5248

**Table 12:** Consistency test results.

Consistency indicators	*A*	*B*_1_	*B*_2_	*B*_3_
*CI*	0.0029	0.0029	0.0019	0.0407
*RI*	0.58	0.90	0.58	0.90
*CR*	0.0050	0.0032	0.0033	0.0452

After the consistency test of each factor is passed, the weight values of each item in the user demand criterion layer of greenhouse pear pollination drone are multiplied by the weight values of the corresponding indicator layer, and the comprehensive weight value of each demand indicator in the entire target demand system can be calculated, as shown Table 13.

**Table 13:** Comprehensive weight of each demand.

Target layer	Criteria layer W	Indicator layer W1-W2		Comprehensive weight
***A***	*B*_1_(Function) *W*_1_ = 0.5949	*C*_*1*_	0.5317	0.3163
		*C*_*2*_	0.1223	0.0728
		*C*_*3*_	0.1856	0.1104
		*C*_*4*_	0.1856	0.1104
	*B*_2_ (Human-Machine Interaction) *W*_2_ = 0.1285	*C*_*5*_	0.1096	0.0141
		*C*_*6*_	0.3091	0.0397
		*C*_*7*_	0.5812	0.0747
	*B*_3_(Security) *W*_3_ = 0.2766	*C*_*8*_	0.0572	0.0158
		*C*_*9*_	0.1101	0.0305
		*C*_*10*_	0.3079	0.0852
		*C*_*11*_	0.5248	0.1452

In this study, the connection between AHP and QFD plays a crucial role. AHP analysis was used to quantify the importance of user requirements. These quantified weights were then incorporated into the QFD House of Quality model. In the QFD model, these weights helped assess the correlation between user requirements and technical features, determining the importance of each technical feature. This process directly translates user needs into technical design priorities, ensuring that the final design meets core user requirements while remaining technically feasible. The integration of AHP and QFD ensures an effective transition from user needs to product technical implementation.

### User demand conversion based on QFD

#### Transforming user needs into technical specifications using QFD

After analyzing and calculating the user requirement weights for the intelligent pollination drone using AHP, we employed Quality Function Deployment (QFD) to translate these requirements into technical specifications. The core of this process is constructing the House of Quality (HOQ), which acts as a bridge between "the voice of the customer" and "the voice of the engineer". The HOQ visually represents the relationship between user needs and product technical features. It also helps calculate the absolute and relative weights of these features and identify potential conflicts in the design process. The HOQ construction process includes the following steps:

Step 1: Constructing the Left Wall of the HOQ. Import the user requirements and their comprehensive weights from Table 13 into the left wall of the HOQ (see Table 16).

Step 2:Constructing the Roof of the HOQ.Based on the technical indicators required to meet the user needs, analyze and expand on the technical indicators for the pollination drone (see Table 14).

**Table 14:** Correspondence between user requirements and technical characteristics.

User primary needs	User secondary needs	Corresponding required skill indicators
Function(*B*_1_)	High pollination efficiency (0.3163)	GPS, ultrasonic positioning
		High-pixel camera
		Optimized pollination path
		Fan-shaped pollination nozzle
		High heat dissipation efficiency
		Eye-catching body design
	Lighting function (0.0728)	LED indicator light
		Eye-catching body design
		GPS, ultrasonic positioning
	Strong environmental adaptability (0.1104)	Waterproof, dustproof and corrosion-resistant
		High heat dissipation efficiency
	Easy to disassemble and assemble (0.1104)	Modular design
		Compact structure design
		Eye-catching body design
Human-Machine Interaction (*B*_2_)	Timely information feedback (0.0141)	Emergency return to flight shutdown
		LED warning light
		Optimize pollination path
		GPS, ultrasonic positioning
	Portability (0.0397)	High-strength lightweight materials
		Modular design
		Compact structure design
	Intelligent operation (0.0747)	GPS, ultrasonic positioning
		Automatic battery replacement
		High-pixel camera
		Automatic pollination tank replenishment
		Automatic cruise obstacle avoidance pollination
Security (*B*_3_)	Easy to identify (0.0158)	Eye-catching fuselage design
		LED warning light
	Battery safety (0.0305)	High heat dissipation efficiency
		Emergency return and stop
		High-strength lightweight materials
		Automatic battery replacement
	Strong structure (0.0852)	High-strength lightweight materials
		Compact structure design
	Emergency response (0.1452)	GPS, ultrasonic positioning
		Emergency return and stop
		Automatic cruise obstacle avoidance and pollination

The results of the product technical characteristics in Table 14 are refined and summarized to obtain the product technical characteristics summary table of greenhouse pear pollination drones, see Table 15. The summary results are imported into the quality house to build the ceiling of the quality house (HOQ), see Table 16.

**Table 15:** Product technical characteristics summary table.

No	Product technical features
1	GPS, ultrasonic positioning
2	High-pixel camera
3	Optimized pollination path
4	Fan-shaped pollination nozzle
5	LED light
6	Automatic refill pollination chamber
7	Automatic cruise obstacle avoidance pollination
8	Waterproof, dustproof and corrosion-resistant
9	High heat dissipation efficiency
10	Modular design
11	High-strength lightweight materials
12	Compact structure design
13	Automatic battery replacement
14	High-purity color contrast
15	Emergency return shutdown

**Table 16:** Greenhouse four seasons pear pollination drone quality house.

Technical requirements User needs		User demand weight	GPS, ultrasonic positioning	High-pixel wide-angle camera	Automatic optimization of pollination path	Fan-shaped pollination nozzle	LED light	Automatic replenishment of pollination chamber	Automatic cruise obstacle avoidance pollination	Waterproof, dustproof and corrosion-resistant	High heat dissipation efficiency	Modular design	High-strength lightweight materials	Compact structure design	Automatic battery replacement	Eye-catching fuselage design	Emergency return stop
Function	High pollination efficiency	0.3163	●	◎	◎	●					●					△	
	Lighting function	0.0728	△				●									◎	
	Strong environmental adaptability	0.1104								●	◎						
	Easy to disassemble and assemble	0.1104										●		△		△	
Human-computer interaction	Timely information feedback	0.0141	◎		△		△										△
	Portability	0.0397										●	◎	△			
	Intelligent operation	0.0747	●	◎				●	●						●		
Security	Easy to identify	0.0158					◎									●	
	Battery safety	0.0305									●		◎		◎		●
	Stable structure	0.0852											●	◎			
	Emergency response	0.1452	◎					△	△						△		●
Absolute weight (*100)			250.57	117.3	96.3	158.15	42.55	80.91	80.91	55.2	206.52	75.05	63.66	40.57	61.02	72.41	92.08
Relative weights (%)			16.78	7.86	6.45	10.6	2.85	5.42	5.42	3.7	13.83	5.03	4.23	2.72	4.09	4.85	6.17

Step 3: Rooms in the House of Quality (HOQ). Analyze and determine the correlation between user needs and product technical characteristics, and use ● (strong correlation), ◎ (medium correlation), and △ (weak correlation) to match the user needs and technical requirements of greenhouse pear pollination drones one by one, where ● = 5, ◎ = 3, △ = 1, and blank space indicates 0 correlation, see Table 16.

Step 4: Basement of the House of Quality (HOQ). Use equations (8) and (9) to calculate the absolute weight and relative weight of the product technical characteristics, and import the calculation results into the House of Quality to build the basement of the House of Quality (HOQ), as shown in Table 16. The specific calculation formula is as follows:

$$W_{j}=\sum_{i=1}^{q}W_{i}P_{ij}$$ 8

$$W_{k}=\frac{W_{j}}{\sum_{i=1}^{q}W_{j}}$$ 9

In the formula:

*W*_*j*_——Absolute weight of the quality characteristics of greenhouse pear pollination drones;

*W*_i_ ——Comprehensive demand weight of greenhouse pear pollination drones users;

*P*_ij_ ——Correlation coefficient between demand weight and quality characteristics;

*W*_k_——Relative weight of the quality characteristics of greenhouse pear pollination drones.

According to the above steps, the quality house model of greenhouse pear pollination drone is obtained as shown in Table 16.

**Fig 3:**
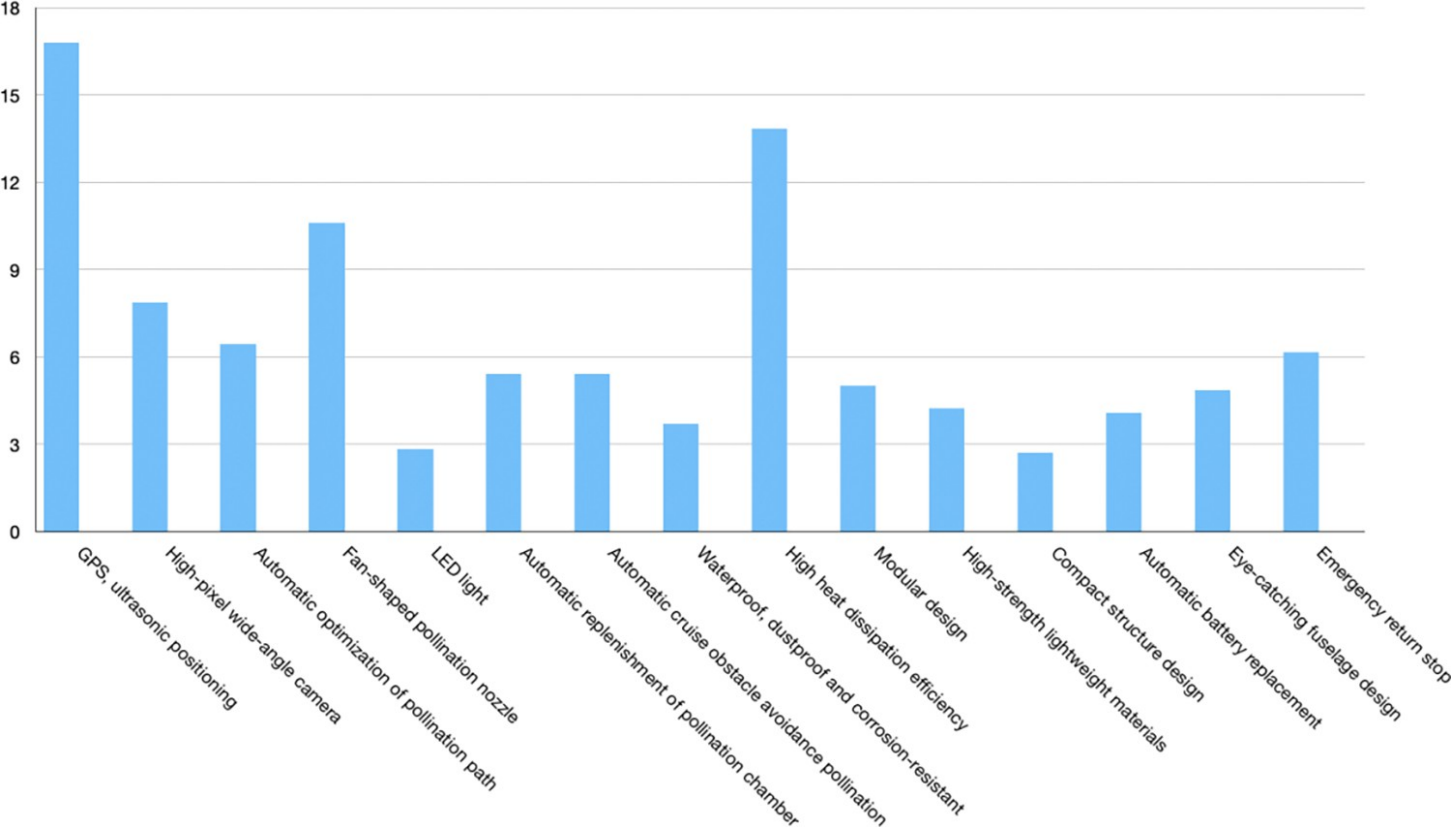
Weight ratio of technical requirements.

According to the QFD theory, the quality house model of the pollination drone is constructed, and the importance weights of the quality characteristics of the pollination drone are obtained. As shown Fig 3. The following conclusions can be drawn from the quality house: in the design of the greenhouse pear pollination drone, GPS, ultrasonic positioning (16.78), high heat dissipation efficiency (13.83), fan-shaped pollination nozzles (10.6), and high-pixel wide-angle cameras (7.86) have the highest weights and are important objects of investigation in the design; automatic optimization of pollination path (6.45), emergency return stop (6.17), automatic replenishment of pollination bins (5.42), automatic cruise obstacle avoidance pollination (5.42), modular design (5.03), and eye-catching fuselage design (4.85) have the second highest weights and are still important objects of investigation in the design; high-strength lightweight materials (4.23), automatic battery replacement (4.09), waterproof, dustproof and corrosion-resistant (3.7), LED lights (2.85), and compact structure design (2.72) are relatively less important and do not require too much attention in the design.

## Results and discussion

The quantitative conclusion of the greenhouse pear pollination drones was drawn based on the QFD quality function deployment theory, focusing on the GPS, ultrasonic positioning (16.78), high heat dissipation efficiency (13.83), fan-shaped pollination nozzle (10.6), high-pixel wide-angle camera (7.86), automatic optimization of pollination path (6.45), emergency return and shutdown (6.17), automatic replenishment of pollination bin (5.42), automatic cruise obstacle avoidance pollination (5.42), modular design (5.03), and eye-catching fuselage design (4.85) of the pollination drone.

**Fig 4:**
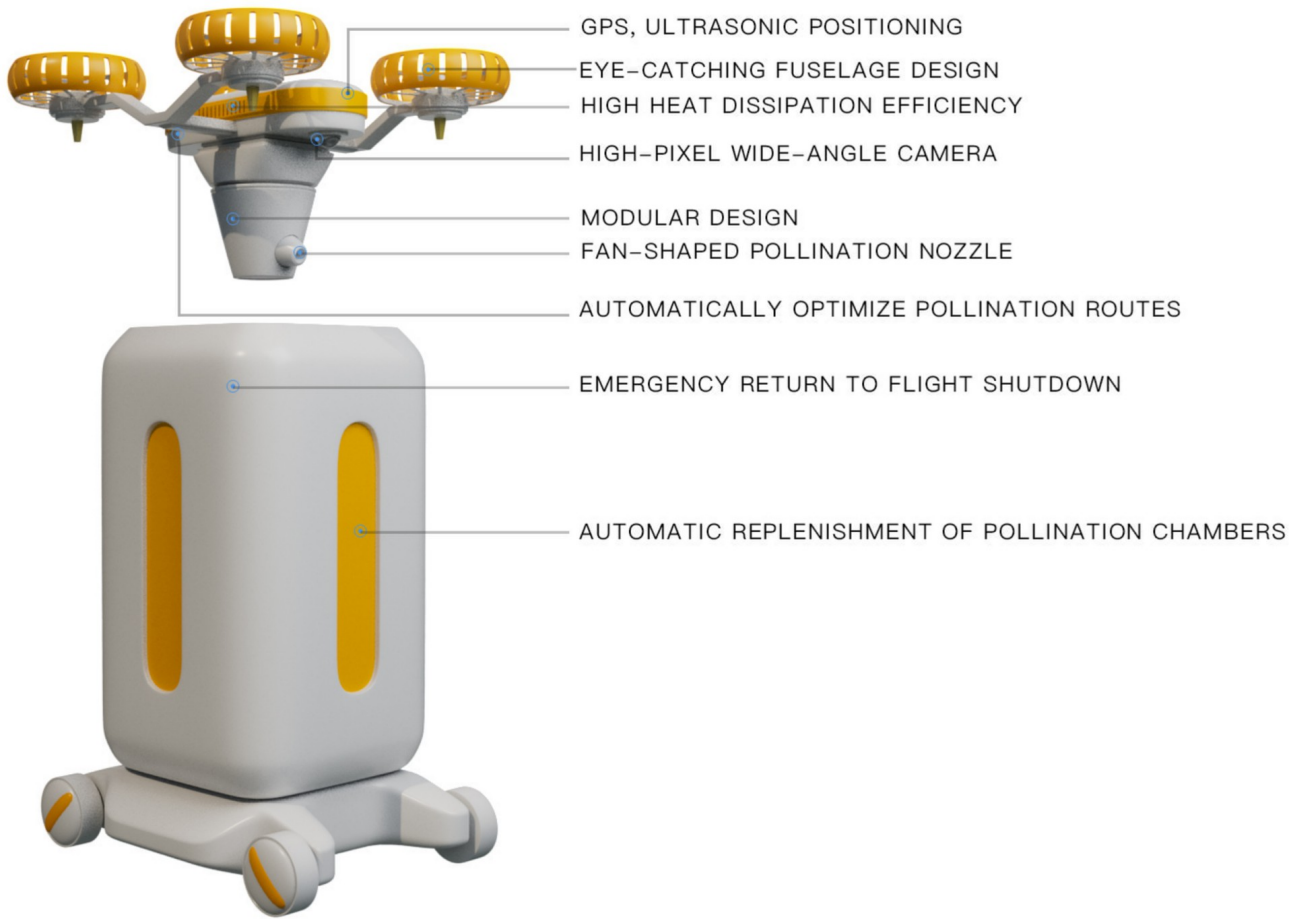
Design rendering of the pollination drone.

Based on the above factors, in the design process of greenhouse pear pollination drones, the precise positioning system of drones is the basic condition to ensure the pollination efficiency of the drones, so it is very important to prioritize it as the primary design element. Due to the poor ventilation of the greenhouse environment and the long-term continuous operation of drones, a lot of heat will be generated, so the heat dissipation design of drones also needs to be included in the important design factors. In order to ensure that the pollen in the drone pollination process can be widely and evenly attached to the stamens, the pollination nozzle of the drone adopts a fan-shaped nozzle to meet the needs. In order to ensure that the drone can accurately and widely identify flowers during the pollination process, a high-definition wide-angle camera is used to meet the needs. In order to meet the automatic pollination function of the drone, the drone can detect the surrounding environment through a high-definition camera and an ultrasonic positioning sensor to automatically optimize the pollination path function and automatically avoid obstacles during the flight. To ensure the safety of the drone, the emergency return and shutdown function of the drone can meet the drone’s self-return and landing when encountering an emergency. The modular design of the drone can simplify the maintenance steps and facilitate fruit farmers to replace damaged parts in time. Since the drone system is relatively small and requires 24-hour contact work, in order to ensure the safety of the operator, the fuselage is designed with a striking appearance and color. The final prototype is shown in Fig 4.

**Fig 5:**
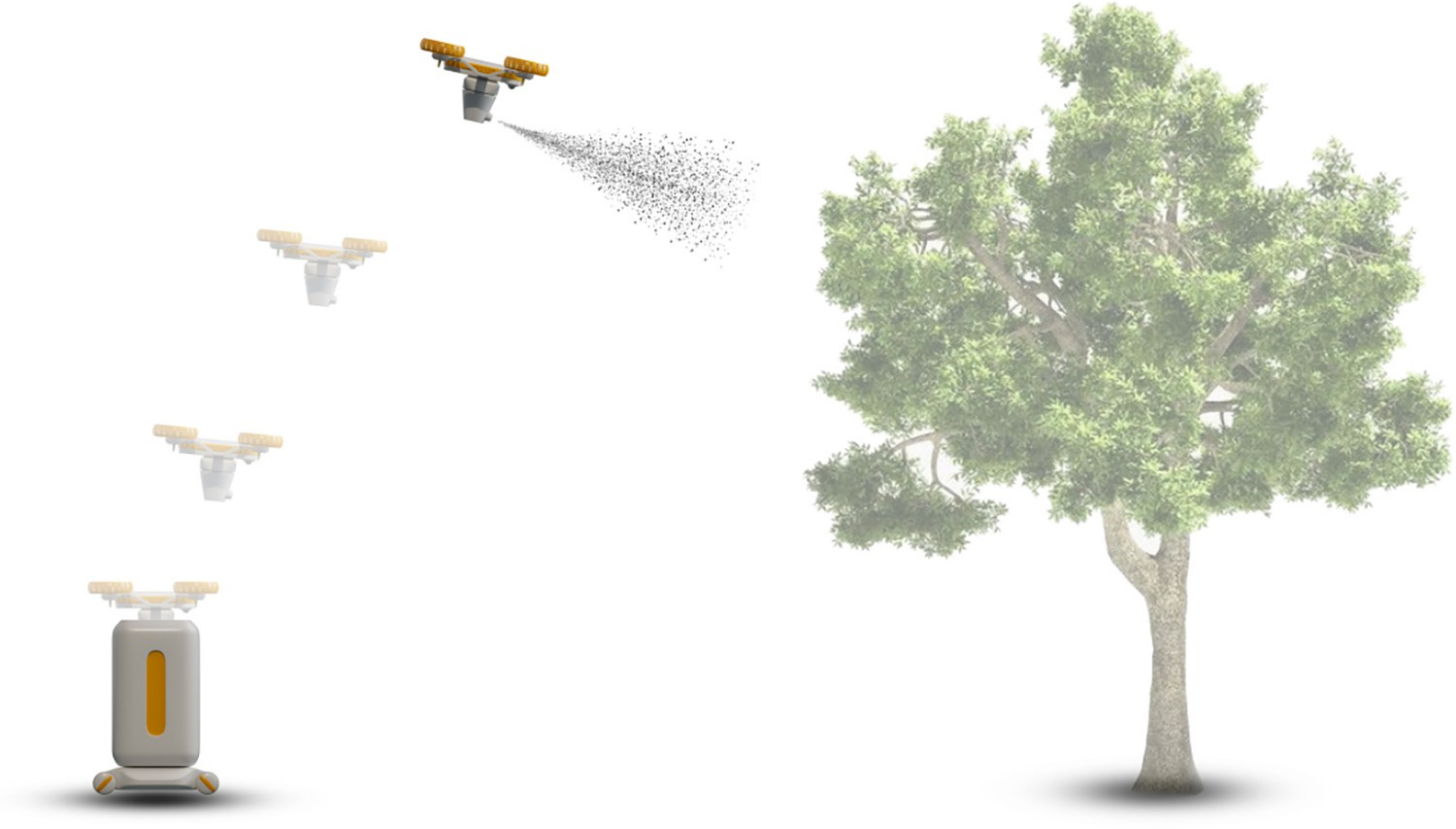
Working principle of pollination drone.

The Automatic replenishment of pollination chamber is the take-off and landing airport of the pollination drone during the pollination process. This equipment can provide power and pollen replenishment for the drone. First, the drone takes off from the Automatic replenishment of pollination chamber, and then pollinates the pear trees in the greenhouse. The drone’s battery and built-in pollination chamber are modularly designed. When the drone’s built-in pollen spraying is finished, the drone will automatically return to the Automatic replenishment of pollination chamber for replenishment or battery replacement, thereby ensuring 24-hour fully automatic pollination and greatly improving pollination efficiency. The pollination process is shown in Fig 5.

This study developed and designed a greenhouse pear pollination drone tailored to the specific needs of pear tree pollination in greenhouse conditions, yielding promising initial results. In future applications, the findings from this research can be leveraged to create pollination drones suited for various crops. However, there are some potential limitations to consider. For instance, variations in temperature and humidity could affect drone performance, and the growth dimensions of different crops might restrict the drone’s operational range. Future research should focus on optimizing design parameters or adding modular features that allow the drone to be adapted for different crop pollination requirements. This modular approach could enhance the drone’s versatility across various greenhouse environments and reduce pollination costs. These considerations offer valuable insights for further research.

## Conclusions

Due to the unique working environment, traditional drones often fail to meet the needs of farmers for pollinating pear trees in greenhouses. The design process for complex agricultural machinery is often fraught with vague information and significant risks, including the involvement of various stakeholders and substantial capital investment. To address this, we conducted an in-depth interdisciplinary study to ensure comprehensive identification of user requirements for greenhouse pear pollination drones. This study utilized Grounded Theory (GT) to gather demand indicators from experts across different fields. These indicators were then weighted using the Analytic Hierarchy Process (AHP), ensuring a user-centered design approach. The Quality Function Deployment (QFD) methodology [36], known for its strong logical and relational foundations, was employed to guide designers in capturing user needs and solving engineering challenges. By integrating qualitative and quantitative findings from GT and AHP, we translated user requirements into technical specifications, quality characteristics, and design elements. This approach provides designers with clear and precise references, thereby reducing the costs associated with the design and development of agricultural machinery. Despite achieving a final design, the sample size for the in-depth interviews was limited, and the drone’s design specifications require further refinement. Future research should expand the sample size and detail the design criteria. Designers should also explore additional user-influencing factors based on different product types and the evolving trends in agricultural machinery to continuously advance product development.
