# Supplementary material for: Evaluation method and design of greenhouse pear pollination drones based on grounded theory and integrated theory
Source: PLoS One. 2024 Oct 29;19(10):e0311297. doi: 10.1371/journal.pone.0311297 (PMC11521276; doi:10.1371/journal.pone.0311297)
Supplement: Attachment — Submitted filename: Response to Reviewers 9.13.docx [file pone.0311297.s001.docx]

Dear Editors and Reviewers,

Thank you very much for taking the time to review this manuscript. We sincerely appreciate your professional review work and valuable feedback. We appreciate the editor and reviewers very much for their positive and constructive comments and suggestions on our manuscript entitled **“Evaluation Method and Design of Greenhouse Pear Pollination Drones Based on Grounded Theory and Integrated Theory”**. These valuable comments helped improve our article and also provided me with valuable experience for my future research. According to your nice suggestions, we have revised the manuscript in depth to make our research more valuable.

We have marked the reviewers’ comments in bold italics and numbered them. Our reply uses normal fonts. In the "Revised Manuscript with Track Changes", we use yellow mark fonts to indicate modifications to the content, and we use red fonts to indicate additions and supplements to the content and pictures. Point-by-point responses to the nice editor and two nice expert reviewers are listed below this letter.

Corresponding author: Tao Wang

E-mail: 260695815@qq.com

**Response to Journal Requirements:**

**1. *Please ensure that your manuscript meets PLOS ONE's style requirements, including those for file naming.***

**Response:** Thank you very much for providing us with the links to the PLOS ONE style templates. We have edited and formatted the revised manuscript according to PLOS ONE's style requirements and file naming requirements.

***2. Please note that funding information should not appear in any section or other areas of your manuscript. We will only publish funding information present in the Funding Statement section of the online submission form. Please remove any funding-related text from the manuscript.***

**Response:** Thank you for your careful reminder. I have removed any funding-related text from the manuscript.

***3. Please state what role the funders took in the study. If the funders had no role, please state: "The funders had no role in study design, data collection and analysis, decision to publish, or preparation of the manuscript."***

***If this statement is not correct you must amend it as needed.***

***Please include this amended Role of Funder statement in your cover letter; we will change the online submission form on your behalf.***

**Response:** Thank you for your patience and careful reminder. I have updated the funder description as requested and included the revised funder role statement in my cover letter.

***4. We note that your Data Availability Statement is currently as follows: All relevant data are within the manuscript and its Supporting Information files.***

***Please confirm at this time whether or not your submission contains all raw data required to replicate the results of your study. Authors must share the “minimal data set” for their submission. PLOS defines the minimal data set to consist of the data required to replicate all study findings reported in the article, as well as related metadata and methods.***

**Response:** Thank you for your patience and careful reminder. I have confirmed that my manuscript contains all the original data required to replicate the research results.

**5. Please review your reference list to ensure that it is complete and correct. If you have cited papers that have been retracted, please include the rationale for doing so in the manuscript text, or remove these references and replace them with relevant current references. Any changes to the reference list should be mentioned in the rebuttal letter that accompanies your revised manuscript. If you need to cite a retracted article, indicate the article’s retracted status in the References list and also include a citation and full reference for the retraction notice.**

**Response:** Thank you for alerting me to this error. I have added the title of the supporting information file to the end of the manuscript and updated the in-text references. Updated references are highlighted in red.

**Response to Reviewers' Comments:**

**Reviewer #1:**

Dear Reviewer #1,

Thank you very much for your thoughtful and positive feedback on my manuscript. I greatly appreciate the time and effort you invested in reviewing my work. Your comments on the clarity of the topic justification, the thoroughness of the literature review, and the robustness of the methodology are especially encouraging. I am pleased to know that you found the data analysis rigorous and the conclusions well-supported. Your recognition of the manuscript's contribution to the field, particularly in providing valuable references for designers and contributing to cost reduction in agricultural machinery design, is very gratifying. Your detailed review has affirmed the scientific rigor and reliability of my research, and I am deeply grateful for your kind words.

Thank you once again for your valuable insights and for acknowledging the strengths of my work. I look forward to contributing further to this field in future studies.

Best regards.

Yours sincerely,

Tao Wang.

Corresponding author: Tao Wang

E-mail: 260695815@qq.com

**Reviewer #2:**

***1. Ensure consistent use of terms like "pollination drones" and "pollination efficiency" throughout the text.*
Response:** Thank you for your insightful feedback on my manuscript. I appreciate your suggestion regarding the consistent use of terms like "pollination drones" and "pollination efficiency." I have carefully reviewed the manuscript to ensure that these terms are used consistently throughout the text, which I believe will enhance the clarity and coherence of the paper.

***2. Some sections, particularly the explanation of Grounded Theory and its application, could be more concise and focused.***

**Response:** Thank you for your detailed and constructive feedback. I have reworked the explanation of grounded theory and its applications to be more concise and focused(Lines131-139).

***3. Discuss potential limitations and areas for future research, such as the scalability of the design or its application to other greenhouse crops.***

**Response:** Thank you for your valuable feedback and for highlighting the importance of discussing potential limitations and areas for future research. We have carefully considered your suggestion and have revised the manuscript accordingly.

In the revised version, we have included a discussion on the potential limitations of the current design, particularly concerning its scalability and the challenges that may arise when applying it to different greenhouse crops. We recognize that while the design has shown promising results in the context of specific crops, further research is needed to assess its effectiveness across a broader range of greenhouse environments and crop types. (Lines439-448)

Additionally, we have identified several areas for future research, including the exploration of modular design approaches. This could allow for greater flexibility and adaptability of the system, making it easier to customize and optimize for different greenhouse conditions and crop types. Modular design could also enhance the scalability of the system, allowing for more efficient implementation across diverse agricultural settings.These directions will help to refine the design further and expand its applicability in the field of agricultural technology.

***4. Clarify the methodology sections by providing more concise explanations of the Grounded Theory and its role in the design process.***

**Response:** Thank you for your valuable feedback on the methodology section. I appreciate your suggestion to provide more concise explanations of Grounded Theory and its role in the design process.

In response to your comments, we have revised the methodology section to offer a clearer and more succinct explanation of Grounded Theory. We have focused on articulating how Grounded Theory was applied in the design process, emphasizing its role in systematically developing design insights based on the data collected. These revisions aim to enhance the clarity of the methodology and ensure that its application is easily understood by the readers(Lines139-141).

***5. Ensure the logical flow of sections, particularly when discussing the integration of AHP and QFD.***

**Response:** Thank you for your constructive feedback on ensuring the logical flow of the sections, particularly regarding the integration of AHP and QFD.

In response to your suggestion, I have carefully reviewed and revised the manuscript to improve the logical flow between the sections discussing the integration of the Analytic Hierarchy Process (AHP) and Quality Function Deployment (QFD). I have reorganized the content to ensure a smoother transition between these topics, clearly outlining how AHP is used to prioritize design criteria and how these priorities are subsequently integrated into the QFD framework (Lines324-331).

***6. Consider using more visual aids, such as diagrams or flowcharts, to illustrate complex processes or design frameworks.***

**Response:** We sincerely appreciate the reviewer’s valuable comments and apologize for the lack of clarity and precision in our manuscript, which may have led to some misunderstanding. To provide a clearer presentation of the overall research process and content, we have summarized the main application steps of the GT, AHP, and QFD methods utilized in this study and illustrated them in a visual flowchart (as shown in Figure 2). In the QFD analysis section, to offer a more intuitive understanding of the weight distribution of the design criteria, we have created Figure 3. Additionally, we have renumbered all figures and tables throughout the manuscript for consistency.

***7. Include more context on how the proposed drone design compares with existing pollination methods and technologies.***

**Response:** Thank you for your suggestion to include more context on how the proposed drone design compares with existing pollination methods and technologies.

In response to your feedback, we have revised the manuscript to provide additional context that highlights the comparative advantages of the proposed drone design. Specifically, I have expanded the discussion to include a comparison with traditional pollination methods, such as manual pollination and the use of natural pollinators, as well as existing technological solutions. This comparison underscores the unique benefits of the proposed design, including its potential for higher efficiency, scalability, and adaptability to different crop types and greenhouse environments（Lines81-87）.

We appreciate your insightful suggestion, which has allowed me to further strengthen the manuscript by offering a clearer perspective on the innovation and significance of the proposed design.

We have tried our best to improve the manuscript, and made some changes marked in blue and red in the revised paper, but they will not affect the content and framework of the paper. We express our sincere thanks to the editor and reviewers for their enthusiastic work and hope that the revisions will be accepted. Thanks again for your valuable and constructive comments and suggestions.

Best regards.

Yours sincerely,

Tao Wang.

Corresponding author: Tao Wang

E-mail: 260695815@qq.com
