# Supplementary material for: Evaluation method and design of greenhouse pear pollination drones based on grounded theory and integrated theory
Source: PLoS One. 2024 Oct 29;19(10):e0311297. doi: 10.1371/journal.pone.0311297 (PMC11521276; doi:10.1371/journal.pone.0311297)
Supplement: Peer review history

# Peer review: Decision Letter 0

**DOI:** 10.1371/journal.pone.0311297.r001

**Authors:** Tariq Umar

6 Aug 2024

PONE-D-24-27533Evaluation Method and Design of Greenhouse Pear Pollination Drones Based on Grounded Theory and Integrated TheoryPLOS ONE

Dear Dr. Wang,

Thank you for submitting your manuscript to PLOS ONE. After careful consideration, we feel that it has merit but does not fully meet PLOS ONE’s publication criteria as it currently stands. Therefore, we invite you to submit a revised version of the manuscript that addresses the points raised during the review process.

Please submit your revised manuscript by Sep 20 2024 11:59PM. If you will need more time than this to complete your revisions, please reply to this message or contact the journal office at plosone@plos.org. When you're ready to submit your revision, log on to https://www.editorialmanager.com/pone/ and select the 'Submissions Needing Revision' folder to locate your manuscript file.

Please include the following items when submitting your revised manuscript:

- A rebuttal letter that responds to each point raised by the academic editor and reviewer(s). You should upload this letter as a separate file labeled 'Response to Reviewers'.
- A marked-up copy of your manuscript that highlights changes made to the original version. You should upload this as a separate file labeled 'Revised Manuscript with Track Changes'.
- An unmarked version of your revised paper without tracked changes. You should upload this as a separate file labeled 'Manuscript'.

If you would like to make changes to your financial disclosure, please include your updated statement in your cover letter. Guidelines for resubmitting your figure files are available below the reviewer comments at the end of this letter.

If applicable, we recommend that you deposit your laboratory protocols in protocols.io to enhance the reproducibility of your results. Protocols.io assigns your protocol its own identifier (DOI) so that it can be cited independently in the future. For instructions see: https://journals.plos.org/plosone/s/submission-guidelines#loc-laboratory-protocols. Additionally, PLOS ONE offers an option for publishing peer-reviewed Lab Protocol articles, which describe protocols hosted on protocols.io. Read more information on sharing protocols at https://plos.org/protocols?utm_medium=editorial-email&utm_source=authorletters&utm_campaign=protocols.

We look forward to receiving your revised manuscript.

Kind regards,

Tariq Umar, PhD, CEng, EUR ING, MICE, FHEA

Academic Editor

PLOS ONE

Journal Requirements:

When submitting your revision, we need you to address these additional requirements.

1. Please ensure that your manuscript meets PLOS ONE's style requirements, including those for file naming. The PLOS ONE style templates can be found at 

https://journals.plos.org/plosone/s/file?id=wjVg/PLOSOne_formatting_sample_main_body.pdf and 

https://journals.plos.org/plosone/s/file?id=ba62/PLOSOne_formatting_sample_title_authors_affiliations.pdf

2. Please note that funding information should not appear in any section or other areas of your manuscript. We will only publish funding information present in the Funding Statement section of the online submission form. Please remove any funding-related text from the manuscript.

3. Thank you for stating the following financial disclosure: 

   "Educational Science Planning Project of Henan Province, China. Project number: 2024YB0265."

Please state what role the funders took in the study.  If the funders had no role, please state: "The funders had no role in study design, data collection and analysis, decision to publish, or preparation of the manuscript." 

If this statement is not correct you must amend it as needed. 

Please include this amended Role of Funder statement in your cover letter; we will change the online submission form on your behalf.

4. We note that your Data Availability Statement is currently as follows: All relevant data are within the manuscript and its Supporting Information files.

Please confirm at this time whether or not your submission contains all raw data required to replicate the results of your study. Authors must share the “minimal data set” for their submission. PLOS defines the minimal data set to consist of the data required to replicate all study findings reported in the article, as well as related metadata and methods (https://journals.plos.org/plosone/s/data-availability#loc-minimal-data-set-definition).

For example, authors should submit the following data:

- The values behind the means, standard deviations and other measures reported;

- The values used to build graphs;

- The points extracted from images for analysis.

Authors do not need to submit their entire data set if only a portion of the data was used in the reported study.

If your submission does not contain these data, please either upload them as Supporting Information files or deposit them to a stable, public repository and provide us with the relevant URLs, DOIs, or accession numbers. For a list of recommended repositories, please see https://journals.plos.org/plosone/s/recommended-repositories.

If there are ethical or legal restrictions on sharing a de-identified data set, please explain them in detail (e.g., data contain potentially sensitive information, data are owned by a third-party organization, etc.) and who has imposed them (e.g., an ethics committee). Please also provide contact information for a data access committee, ethics committee, or other institutional body to which data requests may be sent. If data are owned by a third party, please indicate how others may request data access.

5. Please review your reference list to ensure that it is complete and correct. If you have cited papers that have been retracted, please include the rationale for doing so in the manuscript text, or remove these references and replace them with relevant current references. Any changes to the reference list should be mentioned in the rebuttal letter that accompanies your revised manuscript. If you need to cite a retracted article, indicate the article’s retracted status in the References list and also include a citation and full reference for the retraction notice.

Additional Editor Comments:

Your paper is now reviewed by two reviewers. Please see their comments and revise your paper accordingly.

[Note: HTML markup is below. Please do not edit.]

Reviewers' comments:

Reviewer's Responses to Questions

**Comments to the Author**

1. Is the manuscript technically sound, and do the data support the conclusions?

The manuscript must describe a technically sound piece of scientific research with data that supports the conclusions. Experiments must have been conducted rigorously, with appropriate controls, replication, and sample sizes. The conclusions must be drawn appropriately based on the data presented. 

Reviewer #1: Yes

Reviewer #2: Yes

**********

2. Has the statistical analysis been performed appropriately and rigorously? 

Reviewer #1: I Don't Know

Reviewer #2: N/A

**********

3. Have the authors made all data underlying the findings in their manuscript fully available?

The PLOS Data policy requires authors to make all data underlying the findings described in their manuscript fully available without restriction, with rare exception (please refer to the Data Availability Statement in the manuscript PDF file). The data should be provided as part of the manuscript or its supporting information, or deposited to a public repository. For example, in addition to summary statistics, the data points behind means, medians and variance measures should be available. If there are restrictions on publicly sharing data—e.g. participant privacy or use of data from a third party—those must be specified.

Reviewer #1: Yes

Reviewer #2: Yes

**********

4. Is the manuscript presented in an intelligible fashion and written in standard English?

PLOS ONE does not copyedit accepted manuscripts, so the language in submitted articles must be clear, correct, and unambiguous. Any typographical or grammatical errors should be corrected at revision, so please note any specific errors here.

Reviewer #1: Yes

Reviewer #2: Yes

**********

5. Review Comments to the Author

Please use the space provided to explain your answers to the questions above. You may also include additional comments for the author, including concerns about dual publication, research ethics, or publication ethics. (Please upload your review as an attachment if it exceeds 20,000 characters)

Reviewer #1: The justification for the selected topic is clear and well-articulated. The literature review and methodology are meticulously developed, demonstrating a thorough understanding of the subject matter. The chosen methodology is not only well-justified but also logically structured, providing a robust framework for the research.

There is a high degree of accuracy in outlining the processes for data collection and analysis. The paper exemplifies a technically sound piece of scientific research, with data that robustly supports the conclusions. The analysis is conducted rigorously, incorporating appropriate controls, replication, and sample sizes, ensuring the reliability and validity of the findings.

The conclusions drawn are appropriate and well-supported by the presented data. The statistical analysis is performed rigorously, adhering to high standards of precision and thoroughness. Additionally, the authors have ensured transparency by making all data underlying the findings fully available, facilitating reproducibility and further scrutiny by other researchers.

The manuscript is presented in an intelligible fashion, adhering to standard English, which enhances its accessibility and readability. The approach taken in this manuscript offers clear and precise references for designers, significantly contributing to the reduction of costs associated with the design and development of agricultural machinery. The results provide a solid foundation for subsequent studies, paving the way for future research in this area.

Overall, this paper stands out for its methodical approach, thorough analysis, and clear presentation, making it a valuable contribution to the field. The meticulous attention to detail in the justification of the topic, literature review, methodology, and data analysis underscores the paper's scientific rigour and reliability.

Reviewer #2: The manuscript is generally presented in an intelligible fashion and written in standard English, but there are areas for improvement: 1.Ensure consistent use of terms like "pollination drones" and "pollination efficiency" throughout the text. 2. Some sections, particularly the explanation of Grounded Theory and its application, could be more concise and focused.3. Discuss potential limitations and areas for future research, such as the scalability of the design or its application to other greenhouse crops. 4. Clarify the methodology sections by providing more concise explanations of the Grounded Theory and its role in the design process. 5. Ensure the logical flow of sections, particularly when discussing the integration of AHP and QFD. 6. Consider using more visual aids, such as diagrams or flowcharts, to illustrate complex processes or design frameworks. 7. Include more context on how the proposed drone design compares with existing pollination methods and technologies.

Overall Recommendation: The manuscript presents a valuable and innovative approach to designing greenhouse pollination drones. With minor revisions for clarity and consistency, it will make a strong contribution to the field of agricultural technology.

**********

6. PLOS authors have the option to publish the peer review history of their article (what does this mean?). If published, this will include your full peer review and any attached files.

If you choose “no”, your identity will remain anonymous but your review may still be made public.

**Do you want your identity to be public for this peer review?** For information about this choice, including consent withdrawal, please see our Privacy Policy.

Reviewer #1: No

Reviewer #2: No

**********

[NOTE: If reviewer comments were submitted as an attachment file, they will be attached to this email and accessible via the submission site. Please log into your account, locate the manuscript record, and check for the action link "View Attachments". If this link does not appear, there are no attachment files.]

While revising your submission, please upload your figure files to the Preflight Analysis and Conversion Engine (PACE) digital diagnostic tool, https://pacev2.apexcovantage.com/. PACE helps ensure that figures meet PLOS requirements. To use PACE, you must first register as a user. Registration is free. Then, login and navigate to the UPLOAD tab, where you will find detailed instructions on how to use the tool. If you encounter any issues or have any questions when using PACE, please email PLOS at figures@plos.org. Please note that Supporting Information files do not need this step.

# Author comment: Author response to Decision Letter 0

**DOI:** 10.1371/journal.pone.0311297.r002

13 Sep 2024

Dear Editors and Reviewers,

Thank you very much for taking the time to review this manuscript. We sincerely appreciate your professional review work and valuable feedback. We appreciate the editor and reviewers very much for their positive and constructive comments and suggestions on our manuscript entitled “Evaluation Method and Design of Greenhouse Pear Pollination Drones Based on Grounded Theory and Integrated Theory”. These valuable comments helped improve our article and also provided me with valuable experience for my future research. According to your nice suggestions, we have revised the manuscript in depth to make our research more valuable.

We have marked the reviewers’ comments in bold italics and numbered them. Our reply uses normal fonts. In the "Revised Manuscript with Track Changes", we use yellow mark fonts to indicate modifications to the content, and we use red fonts to indicate additions and supplements to the content and pictures. Point-by-point responses to the nice editor and two nice expert reviewers are listed below this letter.

Corresponding author: Tao Wang

E-mail: 260695815@qq.com

Response to Journal Requirements:

1. Please ensure that your manuscript meets PLOS ONE's style requirements, including those for file naming. 

Response: Thank you very much for providing us with the links to the PLOS ONE style templates. We have edited and formatted the revised manuscript according to PLOS ONE's style requirements and file naming requirements.

2. Please note that funding information should not appear in any section or other areas of your manuscript. We will only publish funding information present in the Funding Statement section of the online submission form. Please remove any funding-related text from the manuscript.

Response: Thank you for your careful reminder. I have removed any funding-related text from the manuscript.

3. Please state what role the funders took in the study. If the funders had no role, please state: "The funders had no role in study design, data collection and analysis, decision to publish, or preparation of the manuscript." 

If this statement is not correct you must amend it as needed. 

Please include this amended Role of Funder statement in your cover letter; we will change the online submission form on your behalf.

Response: Thank you for your patience and careful reminder. I have updated the funder description as requested and included the revised funder role statement in my cover letter.

4. We note that your Data Availability Statement is currently as follows: All relevant data are within the manuscript and its Supporting Information files.

Please confirm at this time whether or not your submission contains all raw data required to replicate the results of your study. Authors must share the “minimal data set” for their submission. PLOS defines the minimal data set to consist of the data required to replicate all study findings reported in the article, as well as related metadata and methods.

Response: Thank you for your patience and careful reminder. I have confirmed that my manuscript contains all the original data required to replicate the research results.

5. Please review your reference list to ensure that it is complete and correct. If you have cited papers that have been retracted, please include the rationale for doing so in the manuscript text, or remove these references and replace them with relevant current references. Any changes to the reference list should be mentioned in the rebuttal letter that accompanies your revised manuscript. If you need to cite a retracted article, indicate the article’s retracted status in the References list and also include a citation and full reference for the retraction notice.

Response: Thank you for alerting me to this error. I have added the title of the supporting information file to the end of the manuscript and updated the in-text references. Updated references are highlighted in red.

Response to Reviewers' Comments:

Reviewer #1:

Dear Reviewer #1,

Thank you very much for your thoughtful and positive feedback on my manuscript. I greatly appreciate the time and effort you invested in reviewing my work. Your comments on the clarity of the topic justification, the thoroughness of the literature review, and the robustness of the methodology are especially encouraging. I am pleased to know that you found the data analysis rigorous and the conclusions well-supported. Your recognition of the manuscript's contribution to the field, particularly in providing valuable references for designers and contributing to cost reduction in agricultural machinery design, is very gratifying. Your detailed review has affirmed the scientific rigor and reliability of my research, and I am deeply grateful for your kind words.

Thank you once again for your valuable insights and for acknowledging the strengths of my work. I look forward to contributing further to this field in future studies.

Best regards.

Yours sincerely,

Tao Wang.

Corresponding author: Tao Wang

E-mail: 260695815@qq.com

Reviewer #2:

1. Ensure consistent use of terms like "pollination drones" and "pollination efficiency" throughout the text.

Response: Thank you for your insightful feedback on my manuscript. I appreciate your suggestion regarding the consistent use of terms like "pollination drones" and "pollination efficiency." I have carefully reviewed the manuscript to ensure that these terms are used consistently throughout the text, which I believe will enhance the clarity and coherence of the paper.

2. Some sections, particularly the explanation of Grounded Theory and its application, could be more concise and focused.

Response: Thank you for your detailed and constructive feedback. I have reworked the explanation of grounded theory and its applications to be more concise and focused(Lines131-139).

3. Discuss potential limitations and areas for future research, such as the scalability of the design or its application to other greenhouse crops.

Response: Thank you for your valuable feedback and for highlighting the importance of discussing potential limitations and areas for future research. We have carefully considered your suggestion and have revised the manuscript accordingly. 

In the revised version, we have included a discussion on the potential limitations of the current design, particularly concerning its scalability and the challenges that may arise when applying it to different greenhouse crops. We recognize that while the design has shown promising results in the context of specific crops, further research is needed to assess its effectiveness across a broader range of greenhouse environments and crop types. (Lines439-448)

Additionally, we have identified several areas for future research, including the exploration of modular design approaches. This could allow for greater flexibility and adaptability of the system, making it easier to customize and optimize for different greenhouse conditions and crop types. Modular design could also enhance the scalability of the system, allowing for more efficient implementation across diverse agricultural settings.These directions will help to refine the design further and expand its applicability in the field of agricultural technology.

4. Clarify the methodology sections by providing more concise explanations of the Grounded Theory and its role in the design process. 

Response: Thank you for your valuable feedback on the methodology section. I appreciate your suggestion to provide more concise explanations of Grounded Theory and its role in the design process.

In response to your comments, we have revised the methodology section to offer a clearer and more succinct explanation of Grounded Theory. We have focused on articulating how Grounded Theory was applied in the design process, emphasizing its role in systematically developing design insights based on the data collected. These revisions aim to enhance the clarity of the methodology and ensure that its application is easily understood by the readers(Lines139-141).

5. Ensure the logical flow of sections, particularly when discussing the integration of AHP and QFD.

Response: Thank you for your constructive feedback on ensuring the logical flow of the sections, particularly regarding the integration of AHP and QFD.

In response to your suggestion, I have carefully reviewed and revised the manuscript to improve the logical flow between the sections discussing the integration of the Analytic Hierarchy Process (AHP) and Quality Function Deployment (QFD). I have reorganized the content to ensure a smoother transition between these topics, clearly outlining how AHP is used to prioritize design criteria and how these priorities are subsequently integrated into the QFD framework (Lines324-331).

6. Consider using more visual aids, such as diagrams or flowcharts, to illustrate complex processes or design frameworks.

Response: We sincerely appreciate the reviewer’s valuable comments and apologize for the lack of clarity and precision in our manuscript, which may have led to some misunderstanding. To provide a clearer presentation of the overall research process and content, we have summarized the main application steps of the GT, AHP, and QFD methods utilized in this study and illustrated them in a visual flowchart (as shown in Figure 2). In the QFD analysis section, to offer a more intuitive understanding of the weight distribution of the design criteria, we have created Figure 3. Additionally, we have renumbered all figures and tables throughout the manuscript for consistency.

7. Include more context on how the proposed drone design compares with existing pollination methods and technologies.

Response: Thank you for your suggestion to include more context on how the proposed drone design compares with existing pollination methods and technologies.

In response to your feedback, we have revised the manuscript to provide additional context that highlights the comparative advantages of the proposed drone design. Specifically, I have expanded the discussion to include a comparison with traditional pollination methods, such as manual pollination and the use of natural pollinators, as well as existing technological solutions. This comparison underscores the unique benefits of the proposed design, including its potential for higher efficiency, scalability, and adaptability to different crop types and greenhouse environments（Lines81-87）.

We appreciate your insightful suggestion, which has allowed me to further strengthen the manuscript by offering a clearer perspective on the innovation and significance of the proposed design.

We have tried our best to improve the manuscript, and made some changes marked in blue and red in the revised paper, but they will not affect the content and framework of the paper. We express our sincere thanks to the editor and reviewers for their enthusiastic work and hope that the revisions will be accepted. Thanks again for your valuable and constructive comments and suggestions.

Best regards.

Yours sincerely,

Tao Wang.

Corresponding author: Tao Wang

E-mail: 260695815@qq.com

Attachment Submitted filename: Response to Reviewers 9.13.docx

# Editor report: Decision Letter 1

**DOI:** 10.1371/journal.pone.0311297.r003

**Authors:** Tariq Umar

18 Sep 2024

Evaluation Method and Design of Greenhouse Pear Pollination Drones Based on Grounded Theory and Integrated Theory

PONE-D-24-27533R1

Dear Dr. Wang,

We’re pleased to inform you that your manuscript has been judged scientifically suitable for publication and will be formally accepted for publication once it meets all outstanding technical requirements.

Within one week, you’ll receive an e-mail detailing the required amendments. When these have been addressed, you’ll receive a formal acceptance letter and your manuscript will be scheduled for publication.

An invoice will be generated when your article is formally accepted. Please note, if your institution has a publishing partnership with PLOS and your article meets the relevant criteria, all or part of your publication costs will be covered. Please make sure your user information is up-to-date by logging into Editorial Manager at Editorial Manager® and clicking the ‘Update My Information' link at the top of the page. If you have any questions relating to publication charges, please contact our Author Billing department directly at authorbilling@plos.org.

If your institution or institutions have a press office, please notify them about your upcoming paper to help maximize its impact. If they’ll be preparing press materials, please inform our press team as soon as possible -- no later than 48 hours after receiving the formal acceptance. Your manuscript will remain under strict press embargo until 2 pm Eastern Time on the date of publication. For more information, please contact onepress@plos.org.

Kind regards,

Tariq Umar, PhD, CEng, EUR ING, MICE, FHEA

Academic Editor

PLOS ONE

Additional Editor Comments (optional):

Thank you for addressing the reviewers comments. The paper is much improved and it is ready for acceptance.

Reviewers' comments:

# Editor report: Acceptance letter

**DOI:** 10.1371/journal.pone.0311297.r004

**Authors:** Tariq Umar

17 Oct 2024

PONE-D-24-27533R1 

PLOS ONE

Dear Dr. Wang, 

I'm pleased to inform you that your manuscript has been deemed suitable for publication in PLOS ONE. Congratulations! Your manuscript is now being handed over to our production team.

At this stage, our production department will prepare your paper for publication. This includes ensuring the following:

* All references, tables, and figures are properly cited

* All relevant supporting information is included in the manuscript submission,

* There are no issues that prevent the paper from being properly typeset

If revisions are needed, the production department will contact you directly to resolve them. If no revisions are needed, you will receive an email when the publication date has been set. At this time, we do not offer pre-publication proofs to authors during production of the accepted work. Please keep in mind that we are working through a large volume of accepted articles, so please give us a few weeks to review your paper and let you know the next and final steps. 

Lastly, if your institution or institutions have a press office, please let them know about your upcoming paper now to help maximize its impact. If they'll be preparing press materials, please inform our press team within the next 48 hours. Your manuscript will remain under strict press embargo until 2 pm Eastern Time on the date of publication. For more information, please contact onepress@plos.org.

If we can help with anything else, please email us at customercare@plos.org.

Thank you for submitting your work to PLOS ONE and supporting open access. 

Kind regards, 

PLOS ONE Editorial Office Staff

on behalf of

Dr Tariq Umar 

Academic Editor

PLOS ONE
